# Reduced *Ribose-5-Phosphate Isomerase A-1* Expression in Specific Neurons and Time Points Promotes Longevity in *Caenorhabditis elegans*

**DOI:** 10.3390/antiox12010124

**Published:** 2023-01-04

**Authors:** Wen-Chi Shen, Chiou-Hwa Yuh, Yu-Ting Lu, Yen-Hung Lin, Tsui-Ting Ching, Chao-Yung Wang, Horng-Dar Wang

**Affiliations:** 1 Institute of Biotechnology, National Tsing Hua University, HsinChu 300044, Taiwan; 2 Institute of Molecular and Genomic Medicine, National Health Research Institutes, Zhunan, Mioali Country 35053, Taiwan; 3 Institute of Biopharmaceutical Sciences, National Yang Ming Chiao Tung University, Taipei 112304, Taiwan; 4 Department of Cardiology, Chang Gung Memory Hospital, Linkou Main Branch, Chang Gung University, Taoyuan 33305, Taiwan; 5 Department of Life Science, National Tsing Hua University, HsinChu 300044, Taiwan

**Keywords:** *ribose-5-phosphate isomerase A* (RPIA), pentose phosphate pathway (PPP), autophagy, AMP activated protein kinase (AMPK), target of rapamycin (TOR), lifespan, *C. elegans*

## Abstract

Deregulation of redox homeostasis is often associated with an accelerated aging process. Ribose-5-phosphate isomerase A (RPIA) mediates redox homeostasis in the pentose phosphate pathway (PPP). Our previous study demonstrated that *Rpi* knockdown boosts the healthspan in *Drosophila*. However, whether the knockdown of *rpia-1*, the *Rpi* ortholog in *Caenorhabditis elegans*, can improve the healthspan in *C. elegans* remains unknown. Here, we report that spatially and temporally limited knockdown of *rpia-1* prolongs lifespan and improves the healthspan in *C. elegans*, reflecting the evolutionarily conserved phenotypes observed in *Drosophila*. Ubiquitous and pan-neuronal knockdown of *rpia-1* both enhance tolerance to oxidative stress, reduce polyglutamine aggregation, and improve the deteriorated body bending rate caused by polyglutamine aggregation. Additionally, *rpia-1* knockdown temporally in the post-developmental stage and spatially in the neuron display enhanced lifespan. Specifically, *rpia-1* knockdown in glutamatergic or cholinergic neurons is sufficient to increase lifespan. Importantly, the lifespan extension by *rpia-1* knockdown requires the activation of autophagy and AMPK pathways and reduced TOR signaling. Moreover, the RNA-seq data support our experimental findings and reveal potential novel downstream targets. Together, our data disclose the specific spatial and temporal conditions and the molecular mechanisms for *rpia-1* knockdown-mediated longevity in *C. elegans*. These findings may help the understanding and improvement of longevity in humans.

## 1. Introduction

Aging is the main process leading to numerous aging-associated diseases, such as neurodegeneration diseases, diabetes, muscular dystrophy, and cancer in elder people [1,2]. Due to the dramatic increment in the elder population, research focusing on the aging process has been increasing. Environmental and physiological interventions, including dietary restriction and reduced temperature or oxygen levels, have been associated with lifespan extension across species. These interventions have been reported to trigger several downstream molecular mechanisms and eventually improve longevity [3].

Among these interventions, dietary restriction, which has prolongevity effects conserved in many species, may broadly affect the numerous downstream molecular mechanisms, including AMP activated protein kinase (AMPK) and target of rapamycin (TOR) signaling pathways [4]. AMPK and TOR pathways are highly associated with nutrient absorption, energy status, and homeostasis [5,6,7]. Previous studies have shown that nutrient deprivation triggers the activation of AMPK and inhibition of the TOR pathway; the synergistic effect of these pathways leads to the induction of the autophagy process and consequently extends lifespan across species [8]. Autophagy is a key process to maintaining cellular homeostasis, whereas failure in autophagic flux perturbs the metabolic balance and exacerbates the aging process [9,10]. It was well reported that the induction of the autophagic flux improves the lifespan in *Drosophila* and *Caenorhabditis elegans* [11,12].

On the other hand, the regulation of redox homeostasis also acts as a key factor in longevity. The redox theory of aging elucidates the relationship between oxidative stress and aging [13]. Moreover, the underlying mechanisms of how redox homeostasis regulates longevity are also well documented [14,15]. The regulation of redox homeostasis includes different defense mechanisms to remove free radicals, such as enzymatic or non-enzymatic antioxidants [16]. These enzymatic antioxidants mainly use nicotinamide adenine dinucleotide phosphate (NADPH), which is primarily produced by the oxidative phase of the pentose phosphate pathway (PPP) [17], as a reducing agent to remove free radicals. Thus, current studies have linked aging with the oxidative phase of PPP since NADPH has been reported to protect species from redox stress, improve oxidative stress tolerance, and extend lifespan [18]. Several approaches to increase NADPH levels, such as the overexpression of *glucose-6-phosphate dehydrogenase* (*G6PD*), *NADPH-generating cytoplasmic malic enzyme* (*Men*), or the knockdown of *ribose-5-phosphate isomerase* (*Rpi*), are all able to extend the lifespan in *Drosophila* [19,20,21,22].

*Ribose-5-phosphate isomerase A (RPIA)*, which is the ortholog of *rpia-1* in *C. elegans*, acts as a rate-limiting enzyme in the pentose phosphate pathway (PPP). Previously, we invented an efficient method to identify the longevity genes in *Drosophila* [23]. Extended by this approach, we identified and demonstrated that reduction in *Rpi,* the *Drosophila* ortholog of *rpia-1* causes lifespan extension and improves healthspan by inducing G6PD activity and elevating NADPH levels to cope with oxidative stress and attenuating polyglutamine toxicity in *Drosophila* [22]. However, whether these phenotypical changes are evolutionarily conserved or not remains unknown. We used *C. elegans* as a model to investigate these phenotypical changes upon *rpia-1* knockdown. Activated autophagy is a well-known longevity-associated mechanism [10]. We and another group revealed that the knockdown of *RPIA* in human cancer cells induces autophagy [24,25]. Hence, we also ask if autophagy induction may contribute to *rpia-1*-mediated lifespan regulation in *C. elegans*.

In this study, we reported that ubiquitous and pan-neuronal knockdown of *rpia-1* result in higher NADPH levels, elevated tolerance to oxidative stress, ameliorated polyglutamine toxicity, and lifespan extension, indicating that *rpia-1* knockdown-mediated longevity and related phenotypical changes may be evolutionarily conserved. In addition, we identified neurons as the target tissue and the young adult stage as the optimal timing for the extended longevity upon *rpia-1* knockdown. Mechanistically, we disclosed that *rpia-1* knockdown-mediated longevity requires the induction of autophagy as well as AMPK activation and TOR inhibition. Furthermore, RNA-seq analysis not only supports our experimental findings but also uncovers more potential downstream target genes, which might be involved in the lifespan regulation upon *rpia-1* knockdown. Together, our results reveal the comprehensive information and molecular mechanisms of how modulation of *rpia-1* regulates longevity and related phenotypes. Those novel findings may advance longevity research to benefit the human lifespan and healthspan.

## 2. Materials and Methods

### 2.1. Strains and Cultivation Conditions

All *C. elegans* strains were cultivated under standard conditions and as described previously [26,27]. The worms were grown at 20 °C on solid nematode growth medium (NGM) agar seeded with *Escherichia coli* (OP50). The following strains were used in this study: N2, wild type (Bristol); AM101, *rmIs110* [*F25B3.3p*::Q40::YFP]; TU3401, *uIs69* [*pCFJ90* (*myo-2p:*:mCherry) + *unc-119p*::*sid-1*]; WM118, *neIs9* [*myo-3:*:HA::RDE-1 + *rol-6(su1006)*]; VP303, *kbIs7* [*nhx-2p*::*rde-1* + *rol-6(su1006)*]; NR222, *kzIs9* [(pKK1260) *lin-26p*::NLS::GFP + (pKK1253) *lin-26p*::*rde-1* + *rol-6(su1006)*]; XE1582, *wpSi11* [*eat-4p*:*:rde-1*::SL2::*sid-1* + *Cbr-unc-119*(+)]; XE1581, *wpSi10* [*unc-17p*::*rde-1*::SL2::*sid-1* + *Cbr-unc-119(+)*]; XE1375, *wpSi1* [*unc-47p*::*rde-1*::SL2::*sid-1* + *Cbr-unc-119(+)*]; XE1474, *wpSi6* [*dat-1p*::*rde-1*::SL2::*sid-1* + *Cbr-unc-119(+)*]; TG38, *aak-2 (gt33)*; RB1206, *rsks-1 (ok1255)*. CRISPR knockout strains *rpia-1 (tm7117)* and *rpia-1 (tm6850)* were outcrossed to N2 wild type animals three times before conducting the experiments. All nematodes were synchronized by bleach and were incubated in M9 buffer without food overnight.

### 2.2. RNA Interference

All RNAi experiments described in this article were induced by feeding assay and as described previously [27,28,29]. Worms were exposed to dsRNA producing *E. coli* HT115 (DE3) strain, which can be induced by isopropyl β-d-1-thiogalactopyranoside (IPTG), starting at the embryonic stage (whole life treatment) or post-developmental stage (adult-only treatment) on solid RNAi plates. The RNAi plates contained 100 μg/mL ampicillin, 10 μg/mL tetracycline, and 1 mM IPTG. RNAi-containing bacteria were grown overnight in LB broth containing 100 μg/mL ampicillin and 10 μg/mL tetracycline at 37 °C. After the overnight incubation, RNAi bacteria were inoculated into new tubes, and dsRNA production was induced by 1 mM IPTG addition. Once the bacteria grew to OD 0.6, they were spread onto the agar plates and left in the hood overnight. These RNAi plates were stored at 4 °C and were used within 2 weeks to ensure knockdown efficiency. The RNAi plasmids were verified by DNA sequencing.

### 2.3. Oxidative Stress Assay

The oxidative stress assay was performed using the protocol proposed by Senchuk et al. [30]. Oxidative stress was induced by 40 mM paraquat (1,1-dimethyl-4,4-bipyridinium dichloride, Sigma-Aldrich, 856177, St. Louis, MO, USA) treatment. Worms were cultivated and synchronized by the bleach assay at 20 °C. Day-0-adult (young adult) worms were transferred onto RNAi plates containing 40 mM paraquat, and dead worms were counted at 0, 15, 19, 23, 27, 39, 43, 47, 51, and 63 h until all worms died. OASIS 2 was used to perform statistical analyses and the *p*-value was calculated by log-rank test.

### 2.4. Quantification of NADP^+^ and NADPH Levels

The NADP^+^ and NADPH levels were measured by using an NADP/NADPH-Glo™ Assay (Promega, G9081, Madison, WI, USA) following the manufacturer’s protocol. Moderate amounts of samples collected in 1.5 mL microcentrifuge tubes were homogenized using a base solution (PBS, 0.2 N NaOH, and 1% SDS). Homogenized samples were transferred to a white-walled 96-well ELISA plate (50 μL per well; two wells per sample). One well of each sample was treated with 25 μL of 0.4 N HCl to measure NADP^+^ levels, and the other was treated with base to measure NADPH levels. After incubating for 15 min, the plate was incubated at room temperature, and HCl/Trizma^®^ solution was added. After incubating the plate at room temperature for 30–60 min, the NADP^+^ and NADPH levels were measured by a luminometer. The definite concentrations of NADP^+^ and NADPH were calculated by a standard curve and normalized to protein concentration.

### 2.5. Lifespan Assay

Before conducting the lifespan assay, all parent worms were cultivated until the day-1-adult stage on solid NGM plates seeded with OP50. At least 100 worms were exposed to bleach buffer and eggs extracted from parents were stored at 18 °C overnight for synchronization as described previously [27,29]. At stage L4 (day 0 of the experiment), the worms were transferred to new plates to start the lifespan assay. Each plate contained 30 worms and the whole experiment was performed at 20 °C. For the first seven days, the worms were transferred every day to separate adults from larvae, and the numbers of censored and dead worms were recorded. After day 7, the worms were transferred and counted every 2 days until all worms died. OASIS 2 was used to perform statistical analyses and the *p*-value was calculated by log-rank test.

### 2.6. Polyglutamine Toxicity

Nematode strain AM101 was used to validate neuronal polyglutamine toxicity. For image analysis, we anesthetized worms using 10 mM sodium azide (NaN_3_) in M9 and mounted the animals on 2% agarose pads dissolved in M9. All worms were grown to the day-5-adult stage, and 10 worms were randomly picked to perform the experiments in triplicates. All magnified neuron images were taken using a ZEISS LSM 800 confocal microscope with a 40x objective; GFP fluorescence quantification was performed with NIS-Elements software. GFP puncta were detected automatically by the software, and statistical analyses were conducted using Prism 8.0.

Body bending assay was conducted on synchronized day-1-adult and day-7-adult stages at 20 °C. Body bending rate was defined as the bending counts of a single worm in 30 s. Each worm was placed in one droplet of M9 and allowed to recover for 1 min. After the recovery, body bends were counted for 30 s by observation. All experiments included 30 animals and were conducted in triplicates. Statistical analyses were performed by using Prism 8.0.

### 2.7. RNA Isolation and Reverse Transcription Followed by Quantitative PCR (RT-qPCR)

At least 500 animals were washed with M9 buffer and lysed in 500 μL Trizol Reagent (APOLO) via standard RNA extraction protocol. The extracted RNA was reverse-transcribed through Reverse Transcription System (Promega, #A3500). We diluted cDNA in a ratio of 1:9 with ddH_2_O to perform quantitative PCR (qPCR) by StepOne Plus Real-time PCR system (Applied Biosystems, cat # 4,376,600, Whaltam, MA, USA) with Fast SYBR™ Green Master Mix (Applied Biosystems, #4385612). The expression levels were determined by the ΔΔCt method and normalized to *act-1* expression levels. All experiments were independently conducted in triplicates and statistical analyses were performed by Prism 8.0 with an unpaired *t*-test.

The following primers were used: *rpia-1,* forward 5′-GTG ACA GAC AAT GGA AAC TTC A-3′, and reverse 5′-ACG CAA CCG ATG AAT AAT CC-3′; *act-1,* forward 5′-ACG ACG AGT CCG GCC CAT CC-3′, and reverse 5′-GAA AGC TGG TGG TGA CGA TGG TT-3′; *lgg-1,* forward 5′-ACC CAG ACC GTA TTC CAG TG-3′, and reverse 5′-ACG AAG TTG GAT GCG TTT TC-3′; *sqst-1,* forward 5′-TGG CTG CTG CAT CAT CCG CT-3′, and reverse 5′-TCA ATC GTG CCG AGA CCG GG-3′.

### 2.8. Protein Extraction and Western Blotting

At least 500–1000 worms were collected and homogenized with whole-cell extract (WCE) lysis buffer (20 mM HEPES [pH 7.5], 75 mM NaCl, 2.5 mM MgCl_2_, 0.1 mM EDTA, 0.5% Triton X-100, 0.1 mM Na_3_VO_4_, 50 mM NaF, protease inhibitor and phosphatase inhibitor), and tetragonal zirconium polycrystal (TZP). Protein extracts were centrifuged at 13,200 rpm for 20 min, and the supernatants were collected. Protein concentration was determined using Bradford assay (Bio-Rad, #5000001, Hercules, CA, USA). After denaturing at 100 °C, the protein samples were run on 10% or 14% polyacrylamide-tris gels (30 μg per well) and transferred onto nitrocellulose (NC) membranes. The membranes were blocked in 5% skim milk or BSA in TBST and incubated with following the antibodies overnight: anti-LC3B (1:1000 in 3% milk in TBST; Novus, St. Charles, MO, USA, #NB100-2220), anti-Ref2P (SQST-1) (1:1000 in 3% milk in TBST; Abcam, Cambrigde, UK, #ab178440), anti-phospho-AMPK (1:1000 in 3% BSA in TBST; Cell Signaling, Danvers, MA, USA, #2535), anti-phospho-S6K (1:1000 in 3% BSA in TBST; Cell Signaling, #9209), anti-beta actin (1:4000 in 3% milk in TBST; GeneTex, #GTX109639). Incubations with the secondary antibody Peroxidase AffiniPure Goat Anti-Rabbit IgG (H+L) (1:10,000 or 1:20,000 diluted in TBST; Jackson Immuno, t# 111-035-003) were performed for 1 h at room temperature. After washing the membranes with TBST three times, the protein bands were detected using ECL.

### 2.9. RNA-Seq Analysis

Samples were collected independently in triplicates, and each sample contained at least 500 worms, which were transferred to new plates every day until the day-5-adult stage. RNA was extracted using RNeasy Kit (Qiagen, #74004). The RNA-seq procedure was conducted by Taiwan Genomics Industry Alliance (TGIA) using 0.1–5 μg of total RNA. Libraries were pooled and sequenced (paired-end, 150 bp) on an Illumina NovaSeq 6000 System.

### 2.10. Developmental Delay Screening

Following a previously proposed protocol [31], we randomly picked five parent worms and split-bleached them onto unseeded solid NGM plates and synchronized the offspring overnight at 20 °C. The synchronized L1 larvae were transferred to bacteria-seeded plates and incubated at 20 °C for 55 h. At this time point, the number of worms at different development stages was counted. Statistical analyses were conducted by using Prism 8.0.

### 2.11. Generation of rpia-1 Overexpression Construct

The promoter and full-length sequences of *rpia-1* were amplified from N2 genomic DNA and cloned into the L3691 plasmid to create the *rpia-1* overexpression construct. The microinjection procedure was conducted by the *C. elegans* Core Facility (CECF). Except for the control and overexpression plasmids, the *myo-2p::tdTomato* plasmid was co-injected as the co-injection marker. At least two independent lines were isolated to obtain stable clones.

The following primers were used: *rpia-1* promoter, forward 5′-ATA TGA GCT CCA ACT GTG TCA CTT CTC TTG TTT C-3′ (*SacI*), and reverse 5′-AGA GAC CGG TTC TGG CGG TTC GCA ATA C-3′ (*AgeI*); *rpia-1* overexpression, forward 5′-ATA TGA GCT CCA ACT GTG TCA CTT CTC TTG TTT C-3′ (*SacI*), and reverse 5′-AGA GAC CGG TGT GCT TCT TGG AAT TGA CAA TTT C-3′ (*AgeI*).

## 3. Results

### 3.1. Knockdown of rpia-1 Exhibits Increased Tolerance to Oxidative Stress, Elevated Levels of NADPH, and Attenuated Polyglutamine Toxicity in C. elegans

**Figure 1:**
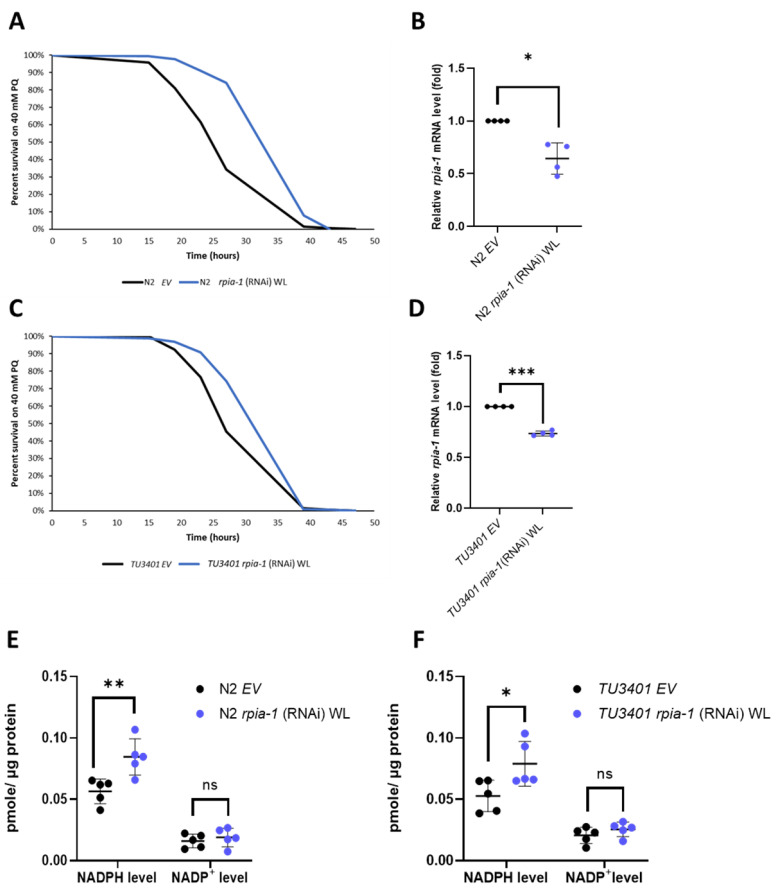
Ubiquitous and pan-neuronal knockdown of *rpia-1* increases oxidative stress tolerance and NADPH levels. (**A**) Whole-life (WL) ubiquitous knockdown of *rpia-1* in wild type N2 resulted in 22.3–32.7% increase in mean survival time under 40 mM paraquat treatment compared with the controls (***, *p* < 0.001, by log-rank test). (**B**) Quantitative RT-qPCR showed reduced *rpia-1* levels (0.6-fold) in worms with whole-life ubiquitous knockdown of *rpia-1* (*, *p* < 0.05, by *t*-test). (**C**) Whole-life pan-neuronal knockdown of *rpia-1* in TU3401 resulted in a 12.7%–14.5% increase in mean survival time under 40 mM paraquat treatment compared with the controls (***, *p* < 0.001, by log-rank test). (**D**) Quantitative RT-qPCR results displayed lowered *rpia-1* levels (0.75-fold) in worms with whole-life pan-neuronal knockdown of *rpia-1* (***, *p* < 0.001, by *t*-test). (**E**,**F**) Both whole-life ubiquitous and pan-neuronal knockdown of *rpia-1* resulted in a 1.5-fold increase in NADPH levels compared with the controls (*, *p* < 0.05; **, *p* < 0.01, by *t*-test).

Down-regulation of *ribose-5-phosphate isomerase* has been shown in *Drosophila* to induce several longevity-associated phenotypes, such as increased tolerance to oxidative stress, elevated levels of NADPH, and alleviated polyglutamine toxicity in neurons [22]. However, whether these effects are evolutionarily conserved remains unclear. Therefore, by utilizing *C. elegans* as another model organism and knocking down *rpia-1*, the ortholog of *ribose-5-phosphate isomerase A* in *C. elegans*, we examined whether the effects of *rpia-1* knockdown from the embryonic stage throughout adulthood (named as whole life (WL) knockdown in this study) in *C. elegans* are conserved as those in *Drosophila*. Similar to our previous findings in *Drosophila*, we observed an elevated tolerance to paraquat-induced oxidative stress ranging from 22.3% to 32.7% in *rpia-1* knockdown N2 worms compared to the wild-type N2 with the empty vector (EV) as the control (Figure 1A,B). According to the results in our previous *Drosophila* study, the reduction in *Rpi* in neurons showed a similar effect as in the *Rpi* mutant with ubiquitous *Rpi* down-regulation [22], we next asked if the enhanced *rpia-1* knockdown in neurons also contributes to oxidative stress resistance. Thus, we conducted an *rpia-1* reduction in the neuron-enhanced strain TU3401 and found similar results to those from an *rpia-1* ubiquitous knockdown in N2. The results revealed that the knockdown of *rpia-1* in the neuron-enhanced strain TU3401 promoted oxidative stress tolerance with an increase ranging from 12.7% to 14.5% compared with the controls (Figure 1C,D). Previous studies have reported that NADPH levels are often increased with the improvement in oxidative stress tolerance due to its involvement in antioxidative systems [32,33]. Consistently, we detected that both ubiquitous and pan-neuronal knockdown of *rpia-1* elevated the levels of NADPH (~1.5-fold) but not those of NADP^+^ (Figure 1E,F). Taken together, these results indicate that the knockdown of *rpia-1* improves oxidative stress tolerance and increases NADPH levels in *C. elegans*, similar to those in *Drosophila*.

**Figure 2:**
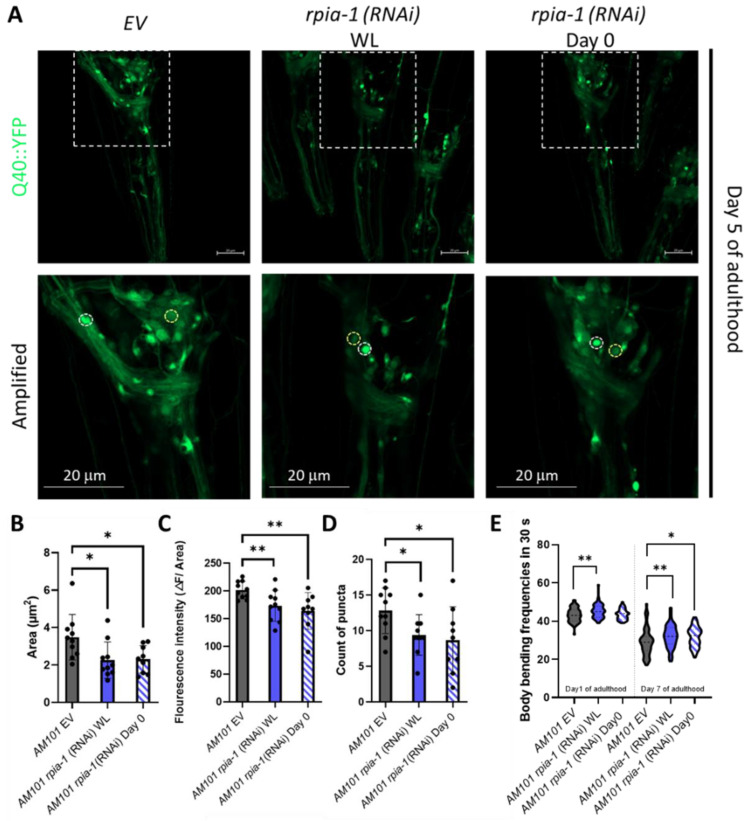
Knockdown of *rpia-1* alleviates polyglutamine toxicity in neurons. (**A**–**D**) Knockdown of *rpia-1* in neuronal polyQ pathogenesis model, AM101, initiated at the whole-life (WL) and at post-developmental stage (Day 0) both reduced puncta area, fluorescence intensity, and puncta count (*, *p* < 0.05; **, *p* < 0.01, by *t*-test). (**E**) The deteriorated body bending rate due to polyQ aggregation may be alleviated by knockdown of *rpia-1* initiated the whole-life (WL) and at post-developmental stage (Day 0) (*, *p* < 0.05; **, *p* < 0.01, by *t*-test and ANOVA).

Polyglutamine (polyQ) aggregation induces oxidative stress by elevating the levels of free radicals and eventually causes damage in the nervous system to trigger neurodegeneration [34]. In addition, the inhibition of NADPH oxidase, which reduces the catalysis of NADPH, ameliorates 43Q protein-induced mitochondrial dysfunction [35,36], implying that elevation of NADPH levels ameliorates polyglutamine-induced neuron toxicity. As we previously reported that the knockdown of *Rpi* increases NADPH levels and alleviates polyglutamine-induced toxicity in *Drosophila* neurons, we next asked whether the knockdown of *rpia-1* can also attenuate polyglutamine-induced neuronal toxicity in *C. elegans* and conducted an *rpia-1* knockdown at two different time points to confirm if the delayed reduction of *rpia-1* can also ameliorate the deterioration in the AM101 strain, which is a neuron-specific polyQ pathogenesis model. Besides the aggregation of polyQ, the previous study also provides evidence that neuronal polyQ aggregation leads to mobility decline during aging. Thus, we further examined if the *rpia-1* knockdown may affect physiological changes, such as body bending rate, in AM101 [37]. The data indicated that the knockdown of *rpia-1* not only reduced polyQ aggregation in nerve rings but also ameliorated the deteriorated body bending rate caused by polyQ aggregation (Figure 2A–E). Overall, these findings suggest that the knockdown of *rpia-1* promotes the longevity-associated phenotypes which could be evolutionarily conserved.

### 3.2. Knockdown of rpia-1 in Specific Time Points and Tissues Displays Extended Lifespan

In addition to the longevity-associated phenotypes, our previous study also revealed that the down-regulation of *Rpi* extends the lifespan in *Drosophila*. Hence, we initially investigated the lifespan of the *rpia-1*-deficient mutant strains by utilizing the *rpia-1* gene partially deleted knockout strains *tm7117* and *tm6850* (Figure S1A), which were generated by the National Bioresource Project in Tokyo [38]. Surprisingly, unlike the *Rpi* mutant flies, the *rpia-1* knockout strains showed no lifespan changes compared with the N2 control (Figure S1B). Additionally, we observed that *rpia-1* knockout strains exhibited a developmental delay in different stages (Figure S1C). These unexpected results may be attributed to the null expression levels of the *rpia-1* knockout strains, in which the CRISPR knockout of *rpia-1* produced *rpia-1* loss-of-function mutations. On the contrary, the *Rpi* mutant in *Drosophila* is generated by P-element insertion resulting in a hypomorphic mutation in *Rpi*. Thus, the different extent of *rpia-1* down-regulation may be a potential factor causing these results.

**Figure 3:**
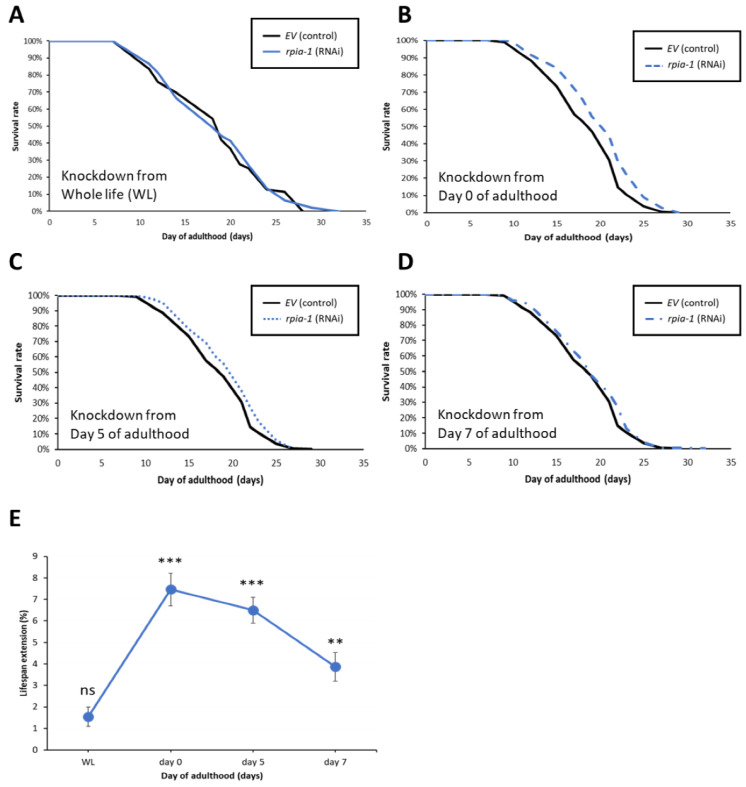
Knockdown of *rpia-1* after developmental stage prolongs lifespan. In these lifespan experiments, solid black line represents the control group, and different blue lines represent the *rpia-1* knockdown groups initiate at different time stage. (**A**) Ubiquitous knockdown of *rpia-1* starting at the embryonic stage caused no lifespan extension, whereas (**B**–**D**) knockdown of *rpia-1* after developmental stage at day 0, day 5, and day 7 resulted in 7.5%, 6.5%, and 3.9% in mean lifespan extension, respectively (**, *p* < 0.01; ***, *p* < 0.001, by log-rank test). (**E**) Among the three different time points of onset of *rpia-1* knockdown, the knockdown initiated on day 0 resulted in the largest amount of lifespan extension.

Instead of using *rpia-1* knockout loss-of-function strains, we assessed the lifespan changes by using an *rpia-1* RNAi knockdown. However, the knockdown of *rpia-1* in the wild type N2 strain from the embryonic stage throughout adulthood (WL) did not show any lifespan change either (Figure 3A) as with the *rpia-1* knockout strains (Figure S1B). This outcome may be caused by two factors: the temporal aspect and the spatial aspect. First, the phenotype of developmental delay in the *rpia-1* knockout mutants implied the possibility that *rpia-1* might play a role during development, whereby the reduced levels of the *rpia-1* transcript during the developmental stage might negatively affect longevity. Second, our previous study revealed that the ubiquitous knockdown of *Rpi* in *Drosophila* only slightly prolongs lifespan, whereas the pan-neuronal knockdown of *Rpi* induces significantly high levels of lifespan extension [22]. Therefore, we further investigated whether *rpia-1* knockdown in a specific time window or specific tissue may mediate lifespan extension in *C. elegans*.

To examine the temporal effect of *rpia-1* knockdown expression and avoid the potentially detrimental effect of *rpia-1* reduction in the developmental stage on lifespan, we postponed the time point of *rpia-1* knockdown initiation to the post-developmental stage and measured the changes in lifespan. The time points we chose were day 0, day 5, and day 7 of adulthood. Day 0 of adulthood indicates the time point of entering the post-developmental stage, which is also known as the young adult (YA) stage. Day 5 of adulthood is the time point after the peak of reproductive development [39], which may be considered menopause in humans. Day 7 of adulthood in our experiments is usually the time when worms start to die. Our results showed that the knockdown of *rpia-1* after the developmental stage in these three post-developmental time points significantly extended mean lifespan by 7.5%, 6.5%, and 3.9%, respectively (Figure 3B–D). Among these three time points, the knockdown of *rpia-1* on day 0 of adulthood resulted in the largest lifespan extension (7–8%) (Figure 3E). In summary, we disclose that the lifespan extension by the *rpia-1* knockdown depends on the post-developmental stage, with the maximal lifespan extension in young adult wild-type N2 worms.

**Figure 4:**
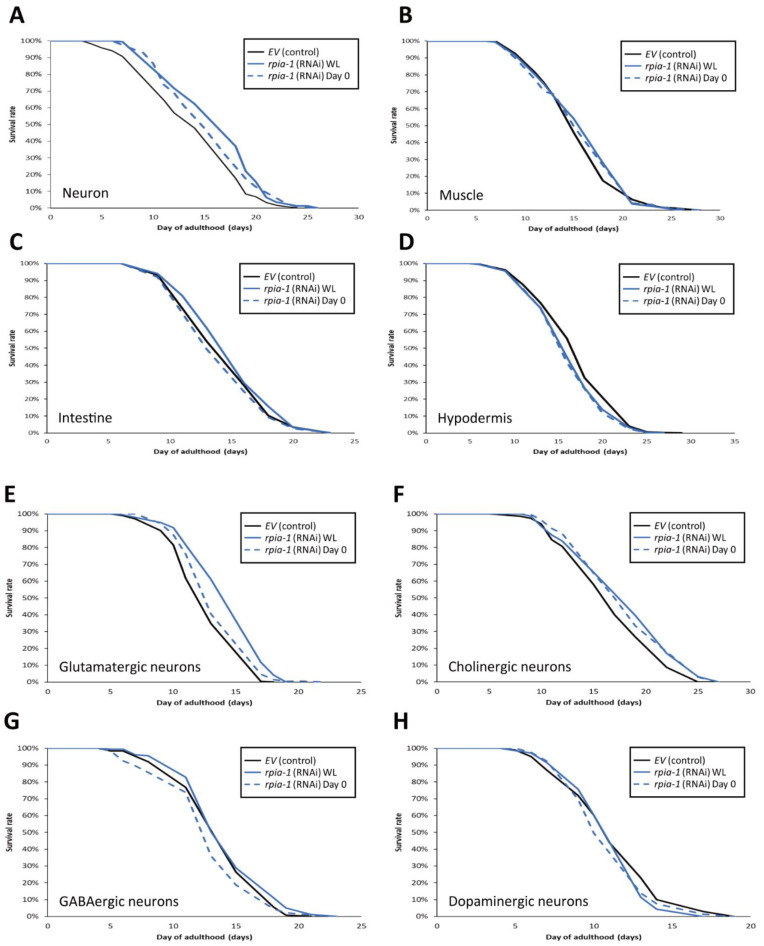
Knockdown of *rpia-1* in glutamatergic or cholinergic neurons prolongs lifespan. In these lifespan experiments, all solid black lines represent control group, and blue lines *rpia-1* knockdown groups. The solid blue lines indicate *rpia-1* knockdown from embryonic stage (WL) and blue dashed lines from day 0 of adulthood. (**A**) Knockdown of *rpia-1* in neurons starting at the embryonic (WL) and post-developmental (Day 0) stages extended the mean lifespan by 13.7% and 5.2%, respectively. (**B**,**C**) Knockdown of *rpia-1* in muscle or intestine showed no effects on lifespan independent of the time of knockdown initiation (since embryonic (WL) or post-developmental (Day 0) stages). (**D**) Knockdown of *rpia-1* in hypodermis shortened the mean lifespan by 4.6% and 5.1% when initiated at the embryonic or post-developmental stages, respectively. (**E**) Knockdown of *rpia-1* in glutamatergic neurons starting from the embryonic or post-developmental stages extended the mean lifespan by 13.4% and 5.7%, respectively. (**F**) Knockdown of *rpia-1* in cholinergic neurons starting from the embryonic or post-developmental stages extended the mean lifespan by 7% and 7%. (**G**,**H**) Knockdown of *rpia-1* in GABAergic or dopaminergic neurons did not affect lifespan regardless of the time of knockdown initiation (since embryonic or post-developmental stages).

To examine whether the spatial aspect of *rpia-1* knockdown expression may play a role in longevity, we reduced the *rpia-1* expression in certain tissues and measured the lifespan changes. Based on our finding that whole-life *rpia-1* ubiquitous knockdown failed to extend lifespan, which might be affected by the spatial aspect, we conducted an *rpia-1* knockdown both in the embryonic stage (WL) and on day 0 of adulthood, respectively. Owing to the fact that the pan-neuronal knockdown of *Rpi* dramatically prolongs the lifespan in *Drosophila*, we looked up the expression patterns of *rpia-1* in WormBase and found that *rpia-1* has been reported to be expressed in the neurons, muscles, intestine, and hypodermis. Hence, we conducted lifespan experiments in four different tissue-specific strains (Table 1). All the strains mentioned below have been used in various lifespan assays and exhibited longevity-associated phenotypes. By reducing the *rpia-1* expression in these strains, we found that worms with pan-neuronal knockdown of *rpia-1* exhibited the highest levels of lifespan extension (13.7%) (Figure 4A), whereas hypodermis-specific *rpia-1* knockdown displayed reduced lifespan (−4.6%) (Figure 4D), yet no changes in muscle or in the intestine (Figure 4B,C). The data indicate that neurons may be at least one of the major target tissues responsible for lifespan extension upon *rpia-1* knockdown in *C. elegans*, which is also similar to our previous finding in *Drosophila*.

Since the worms with pan-neuronal knockdown of *rpia-1* exhibited the largest levels of lifespan extension, we wondered if the reduction in *rpia-1* level in certain specific neurons is sufficient to display lifespan extension. To address this, we conducted lifespan assays in neuron cell-type-specific strains (Table 2) [44] and down-regulated *rpia-1* expression from the embryonic stage (WL) and day 0 of adulthood in those strains to measure the lifespan. Our results indicated that whole-life knockdown of *rpia-1* either in glutamatergic neurons in XE1582 or in cholinergic neurons in XE1581 was sufficient to extend lifespan (Figure 4E,F). In contrast, the knockdown of *rpia-1* in GABAergic neurons in XE1375 or in dopaminergic neurons in XE1474 showed no effect on longevity (Figure 4G,H). Interestingly, the whole-life knockdown of *rpia-1* in glutamatergic neurons in XE1582 exhibited the largest amount of mean lifespan extension (13.4%), similar to the pan-neuronal knockdown of *rpia-1* in TU3401. It may imply that glutamatergic neurons could be the major target neuron cell type in the neuron tissue for lifespan extension by *rpia-1* knockdown. Together, these results indicate that the knockdown of *rpia-1* in glutamatergic neurons or in cholinergic neurons is sufficient to prolong the lifespan in *C. elegans*.

### 3.3. Knockdown of rpia-1 Extends Lifespan by Activating Autophagy and AMPK Pathway and by Inhibiting TOR Pathway

Recently, a positive relationship between the elevation of NADPH levels and lifespan extension has been reported [18,46]. Since NADPH is generated in the oxidative phase of the pentose phosphate pathway (PPP) [47], we previously reported that the deregulation of *Rpi* may constrain the process of PPP from entering the non-oxidative phase. By this limitation, the PPP may be sustained in the oxidative phase and produce more NADPH to improve longevity [22]. Indeed, we also confirmed the elevation of NADPH levels and the lifespan extension in worms by *rpia-1* knockdown (Figure 1E,F and Figure 3), supporting our previous theory.

**Figure 5:**
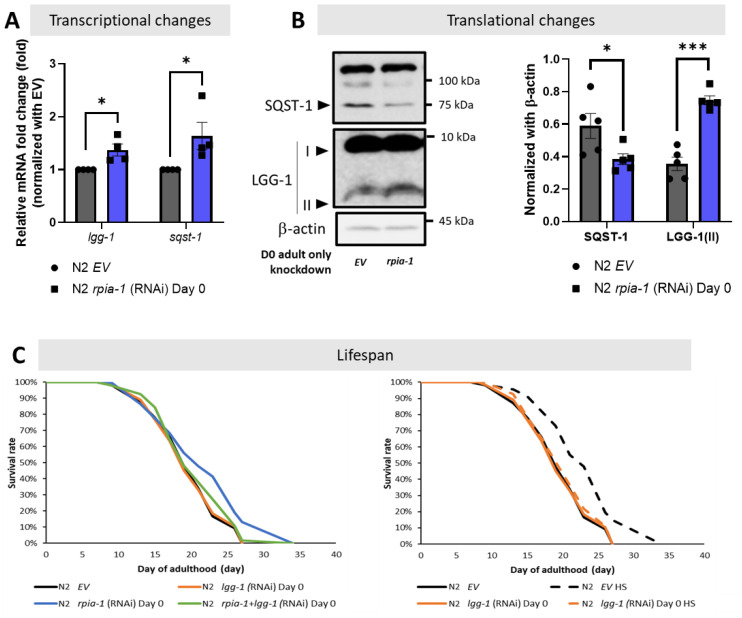
Knockdown of *rpia-1* prolongs lifespan by inducing autophagy. (**A**) By conducting qPCR analysis, the transcriptional levels of autophagy-related marker genes *lgg-1* and *sqst-1* were significantly induced when *rpia-1* was ubiquitously reduced (*, *p* < 0.05; ***, *p* < 0.001, by *t*-test). (**B**) By Western blotting analysis, significantly reduced SQST-1 level and increased LGG-1-II level were detected upon *rpia-1* knockdown ubiquitously (*, *p* < 0.05; ***, *p* < 0.001, by *t*-test). (**C**) (In left panel) Ubiquitous knockdown of *rpia-1* from day 0 of adulthood (blue solid line) extended the mean lifespan by 11.6%, whereas the knockdown of *lgg-1* from day 0 of adulthood (orange solid line) which suppresses autophagy, ablated the *rpia-1* -mediated lifespan extension (green solid line) (***, *p* < 0.001, by log-rank test). (In right panel) Knockdown efficiency of *lgg-1* on lifespan assay was confirmed by *lgg-1* knockdown before hormetic heat shock treatment in wild-type N2 strain, which was reported to extend lifespan through the induction of autophagy. Hormetic heat shock treatment prolonged the mean lifespan by 19.2% (black dashed line), but the lifespan extension was ablated by concomitant *lgg-1* knockdown (orange dashed line) (***, *p* < 0.001, by log-rank test).

Although the balance of redox homeostasis is one of the longevity-associated factors, we asked if any other molecular mechanisms were participating in the *rpia-1* knockdown-mediated longevity. We and another group showed that the down-regulation of *RPIA*, the human ortholog of *rpia-1*, activates autophagy [24,25]. Autophagy induction has been associated with lifespan and healthspan extension [9]. Thus, by following the autophagy guideline and several studies in autophagy, we designed a series of experiments to investigate the levels of autophagy-related genes in *rpia-1* knockdown in the day 0 of adulthood group, since this group of worms extended lifespan the most [9,10,11,12,48,49,50]. For the investigation at the transcriptional level, we validated the mRNA expression levels of *lgg-1* and *sqst-1*, which are two autophagy-related genes represented as markers for mid- and late-autophagy; they are orthologs of human LC3 and SQSTM1/p62, respectively [11,12]. If autophagy is induced, we may find the transcriptional levels of these genes increasing. For the translational aspect, we observed the protein expression of LGG-1/LC3 and SQST-1/SQSTM1/p62. Based on the studies in autophagy, upon induction of autophagy, the LGG-1 precursor is cleaved from the LGG-1-I unlipidated form into the LGG-1-II lipidated form to elongate the phagophores in the mid-stage of autophagy; on the other hand, SQST-1 functions as an autophagosome cargo protein which brings the targeted proteins into autophagosome to be lysed simultaneously, hence the induction of autophagy process leads to SQST-1 degradation [11,12]. From our results, we found that the reduction of *rpia-1* is accompanied by the up-regulation of autophagy-related genes *lgg-1* and *sqst-1* at the transcriptional level (Figure 5A), and with reduced SQST-1 levels and increased LGG-1-II form levels at the translational level (Figure 5B), which both suggest that a reduction in *rpia-1* is associated with activated autophagy. Next, we also conducted lifespan assays in worms with an *rpia-1* knockdown from day 0 of adulthood under an autophagy-reduction background by knockdown of *lgg-1*. We referred to the experimental design from Kumsta et al., in which they concluded that the HSF-1-mediated lifespan extension is directly caused by autophagy induction by blocking *lgg-1*. To confirm whether knockdown of *lgg-1* affected lifespan, they applied hormetic heat shock as a positive control, with which they proved that HSF-1 overexpression can extend lifespan by inducing autophagy priorly, and found that the knockdown of *lgg-1* ablated the lifespan extension caused by HSF-1 overexpression [49]. According to their experimental design, we first confirmed that the autophagy-mediated lifespan extension caused by hormetic heat shock can be ablated by reducing *lgg-1* [49] (Figure 5C, right panel). Under this premise, we then conducted lifespan assays for *rpia-1* knockdown from day 0 of the adulthood group under autophagy-reducing conditions by knockdown of *lgg-1*. In line with our hypothesis, the lifespan extension by *rpia-1* knockdown from day 0 of adulthood is blocked when autophagy was suppressed by *lgg-1* knockdown (Figure 5C, left panel), indicating *rpia-1* knockdown-mediated longevity requires activated autophagy. Taken together, these results suggest that knockdown of *rpia-1* may extend lifespan by inducing autophagy.

**Figure 6:**
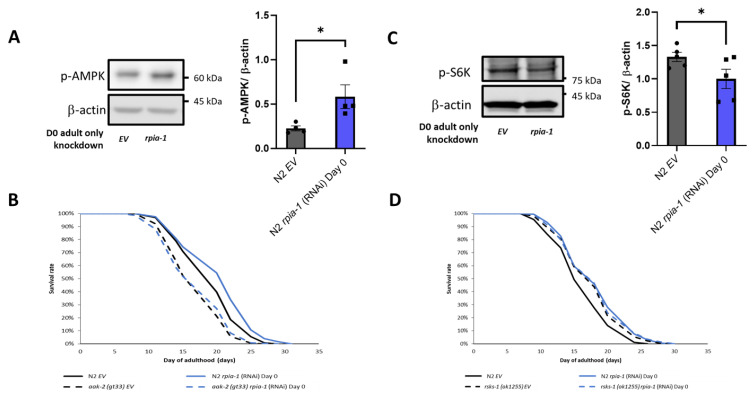
Knockdown of *rpia-1* prolongs lifespan through inducing AMPK pathway and reducing TOR pathway. (**A**) Western blotting results showed that ubiquitous knockdown of *rpia-1* starting at post-developmental stage elevated p-AMPK levels, indicating increased AMPK pathway activity (3-fold increase; *, *p* < 0.05, by *t*-test). (**B**) Ubiquitous knockdown of *rpia-1* from day 0 of adulthood in wild type N2 strain prolonged the mean lifespan by 6.7% (blue solid line); in contrast, ubiquitous knockdown of *rpia-1* in short-lived *aak-2 (gt33)* strain (black dashed line, −10.7%) failed to induce lifespan extension (blue dashed line). (**C**) Western blotting results showed that ubiquitous knockdown of *rpia-1* starting at post-developmental stage reduced levels of p-S6K, a marker of TOR pathway activity (0.8-fold; *, *p* < 0.05, by *t*-test). (**D**) Ubiquitous knockdown of *rpia-1* in wild type N2 strain prolonged the mean lifespan by 11.4% (blue solid line); in contrast, ubiquitous knockdown of *rpia-1* in long-lived *rsks-1 (ok1255)* strain (black dashed line, 7.8%) did not further extend the long-lived lifespan in *rsks-1 (ok1255)* strain (blue dashed line).

According to our data and previous findings, the knockdown of *rpia-1*/*Rpi* extends lifespan associated with elevated NADPH levels in *C. elegans* and in *Drosophila* [22], and *RPIA* knockdown induces autophagy in human colorectal cancer cells [25]. The AMPK pathway and TOR pathway have been shown to regulate the autophagy process in previous studies [51]. These pathways not only regulate lifespan but are also involved in energy homeostasis, which may be related to NADPH generation [52]. Hence, to assess whether *rpia-1* knockdown may prolong lifespan by activating AMPK and inhibiting TOR, we conducted Western blotting analyses, by detecting p-AMPK and p-S6K as the markers for AMPK and TOR pathway activity, respectively, in *rpia-1* RNAi knockdown and control (EV) N2 worms. The results indicated that the knockdown of *rpia-1* from day 0 of adulthood causes a two-fold increase in p-AMPK levels and a reduction in p-S6K levels by 0.75-fold (Figure 6A,C), suggesting that AMPK activation and TOR inhibition may participate in *rpia-1*-knockdown mediated longevity. To confirm the hypothesis, we performed the lifespan analysis for *rpia-1* knockdown from day 0 of adulthood in an *aak-2(gt33)* deletion mutant for the AMPK pathway, for which *aak-2* is the ortholog of human AMP-activated protein kinase catalytic subunit alpha ½, and in the *rsks-1*(*ok1255*) mutant for the TOR pathway, for which *rsks-1* is the ortholog for human ribosomal protein S6 kinase B2, as well as in N2 as the control. The lifespan extension caused by the *rpia-1* knockdown from day 0 of adulthood was blocked in the *aak-2*(*gt33*) mutant (Figure 6B), suggesting that *rpia-1* knockdown-mediated longevity requires *aak-2*. Moreover, the longevity of *rsks-1*(*ok1255*) was not further extended upon *rpia-1* knockdown (Figure 6D), indicating that *rpia-1* knockdown-mediated longevity is involved in reduced TOR signaling. Together, these data support that the knockdown of *rpia-1* prolongs lifespan probably through activating AMPK and inhibiting TOR pathways as well.

### 3.4. RNA Sequencing Analysis Reveals Potential Downstream Target Genes in rpia-1 Knockdown-Mediated Longevity Regulation

To further validate how the knockdown of *rpia-1* activates these longevity-related molecular mechanisms as well as better identify the genes involved in longevity by *rpia-1* knockdown, we chose the two most long-lived strains by *rpia-1* knockdown according to our previous lifespan results (Figure 3 and Figure 4), which are *rpia-1* RNAi knockdown strains in day-0-onset ubiquitous N2 (N2 *rpia-1* (RNAi) Day 0) and in whole-life pan-neuronal TU3401 (TU3401 *rpia-1* (RNAi) WL), together with their control worms for RNA sequencing (RNA-seq) and further analyses to identify the downstream genes and pathways which may lead to improved longevity by *rpia-1* RNAi knockdown.

By using the Ingenuity Pathway Analysis (IPA), we found that both long-lived *rpia-1* knockdown strains showed suppressed organismal death and enhanced cell viability, supporting our findings in longevity regulation (Figure S2A). Furthermore, IPA also revealed that the knockdown of *rpia-1* in neurons reduces glutamate receptor signaling (Figure S2B); hyperexcitable glutamatergic neurons shorten lifespan, supporting our findings that knockdown of *rpia-1* in glutamatergic neurons prolongs lifespan (Figure 4E). Consistent with our results mentioned above (Figure 5), IPA showed that both long-lived *rpia-1* knockdown strains exhibited the induction of several autophagy-related bio-functions as well as the activation of the autophagy process and phagosome formation (Figure S3A,B). Moreover, besides autophagy activation, canonical pathway analysis also showed that several longevity-related pathways, such as the AMPK pathway and sirtuin signaling pathway, were induced (Figure S3B).

**Figure 7:**
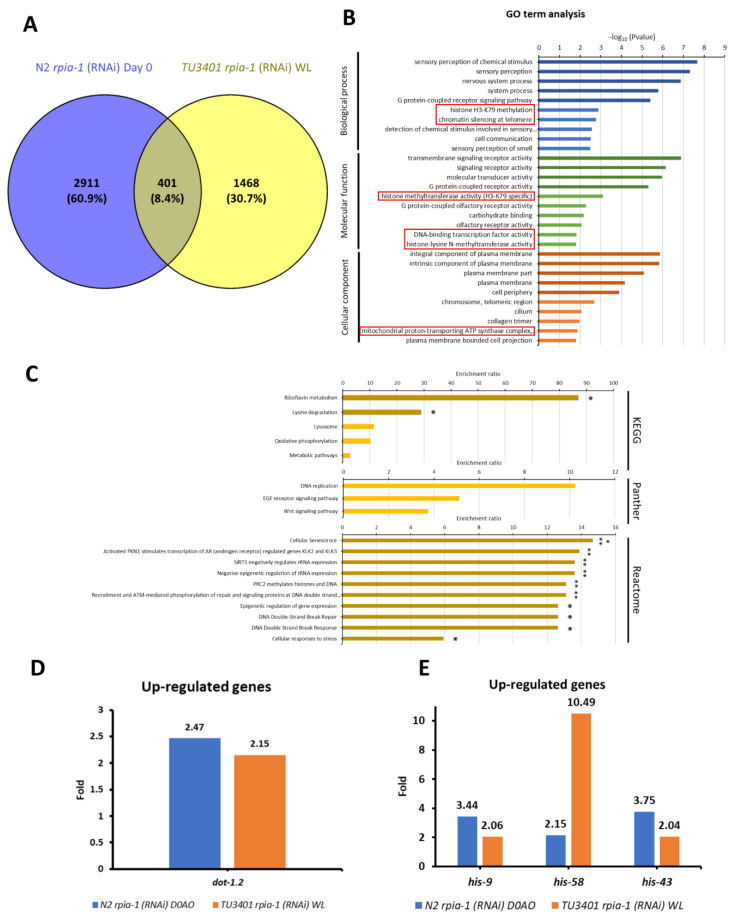
ORA analysis identifies potential up-regulated target genes that may be involved in *rpia-1* knockdown-mediated longevity. Before conducting ORA analysis, we set the threshold for change in gene expression levels to two-fold to filter out genes that showed smaller changes in expression. (**A**) 401 genes were identified as up-regulated in worms with both ubiquitous and pan-neuronal knockdown of *rpia-1*, and these 401 genes were applied to conduct further ORA analysis by WebGestalt. For (**B**,**C**), the data presented are log-transformed *p*-value (FDR corrected) of GO terms or pathways found to be enriched in the up-regulated gene set. (**B**) GO term analysis revealed the gene classes with significant changes. The red frames indicate the classes which were specifically found among the up-regulated gene sets. (**C**) Pathway analyses results including KEGG, Panther, and Reactome. Each pathway enrichment analysis showed the enrichment ratio of the pathway. The bars with darker colors indicated the gene set of pathways was significantly induced. (*, *p* < 0.05; **, *p* < 0.01 by Fisher’s exact test). (**D**) The gene was identified by GO-term analysis. (**E**) The genes identified by GO-term analysis. In (**D**,**E**), blue bars indicate changes in gene expression levels upon ubiquitous *rpia-1* knockdown; orange bars indicate those upon pan-neuronal *rpia-1* knockdown.

In addition to IPA, we also conducted an over-representation analysis (ORA), which is the most broadly applied tool in RNA-seq analysis, to validate potential downstream target genes responsible for the induction of longevity-related molecular mechanisms. Before conducting the analysis, we set the threshold of expression level difference to two-fold for filtering; if the gene expression changes did not reach the threshold for a particular gene, we considered this gene to show no differential gene expression. By comparing the filtered gene sets of long-lived *rpia-1* knockdown strains, we found that 401 genes were up-regulated (Figure 7A) and 264 were down-regulated (Figure S4A). Next, we compared the gene ontology (GO) term analysis results of these 401 up-regulated and 264 down-regulated genes and identified the gene classes in each group for further analysis. We uncovered that the histone H3-K79 methylation may be an up-regulation-specific class (Figure 7B); on the other hand, fucosylation and synapse-related gene classes, which have not been reported to be associated with longevity, may be the down-regulation-specific classes (Figure S4B).

Histone H3-K79 methylation has been reported to be associated with longevity. Previous studies not only verified that the induction of H3-K79 methylation acts as a downstream process of AMPK-induced longevity, but they also revealed that the inhibition of H3-K79 methylation by inhibiting histone methyltransferases may interfere with longevity [53,54,55]. The histone methyltransferase in *C. elegans* is encoded by *dot-1.2*, which was up-regulated in the long-lived *rpia-1* knockdown strains (Figure 7D). This may support our finding that the knockdown of *rpia-1* mediates lifespan through activating AMPK and might trigger H3-K79 methylation by increasing *dot-1.2* expression.

Along with the GO term analysis, we also conducted pathway analysis in 401 up-regulated genes and 264 down-regulated genes based on ORA. By KEGG, Panther, and Reactome pathway analyses, we found that histone genes, including *his-9*, *his-58*, and *his-43*, were up-regulated in long-lived *rpia-1* knockdown strains (Figure 7C,E). Previous studies and our studies have demonstrated that the elevation of histone gene expression extends the lifespan in both *Drosophila* and *C. elegans* [56,57]. Moreover, the study in *Drosophila* mentioned that histone overexpression extends lifespan through the sirtuin signaling pathway, which also echoes our IPA results, suggesting that *his-9*, *his-58*, and *his-43* might be potential downstream target genes in *rpia-1* knockdown-mediated lifespan regulation. Unlike up-regulated gene sets, pathway analysis in down-regulated gene sets exhibited a weak connection to longevity. Three genes were identified by pathway analysis, including *gmd-2*, *ZK697.8*, and *scl-14* (Figure S4C,D). Although *gmd-2* is involved in fructose and mannose metabolism, and *scl-14* is involved in the PI3 kinase pathway, they were not reported to be associated with longevity. *ZK697.8* encodes the protein transthyretin, which is associated with neurodegenerative diseases and has been reported to negatively regulate lifespan in *Drosophila* [58]. Altogether, RNA-seq analyses not only support our results mentioned above but also uncover more potential downstream target genes worthy of further investigation.

## 4. Discussion

**Figure 8:**
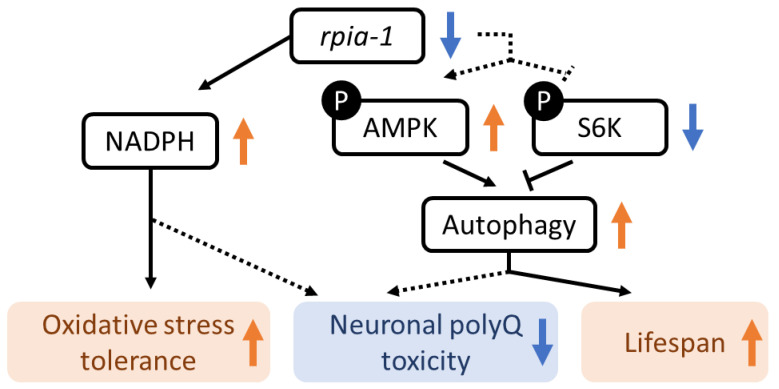
The mechanism of *rpia-1*-mediated lifespan regulation and oxidative stress response in *C. elegans.* The down-regulation of *rpia-1* results in the elevation of NADPH level, which also benefits oxidative stress tolerance improvement. Moreover, according to previous studies, the elevation of NADPH might benefit the amelioration of aggregated neuronal polyQ implying that *rpia-1* reduction-induced neuronal polyQ aggregation alleviation might be attributed to NADPH level. Still, further experiment is required. In addition, *rpia-1* reduction positively regulates the AMPK pathway and negatively regulates the TOR pathway, which induces autophagy and eventually leads to lifespan extension in *C. elegans.* Besides lifespan extension, the neuronal polyQ aggregation might also be alleviated by *rpia-1*-mediated autophagy induction.

In this study, we found that the reduction in *rpia-1* improves longevity, enhances oxidative stress tolerance, and alleviates neuronal polyglutamine toxicity in *C. elegans*. Furthermore, we provided evidence that this phenomenon of lifespan and healthspan regulation may be attributed to the elevation of NADPH levels, activation of autophagy and AMPK pathways, and inhibition of the TOR pathway (Figure 8). In addition, we confirmed that the knockdown of *rpia-1* in certain spatial and temporal conditions can lead to the largest amount of lifespan extension. Although we were not able to detect lifespan extension in *rpia-1* CRISPR knockout strains, our results revealed that ubiquitous knockdown of *rpia-1* in the post-developmental stage prolongs lifespan. Despite our previous study showing that the *Drosophila* hypomorph *Rpi* mutant displays an extended lifespan [22], *rpia-1* knockout mutants did not exhibit lifespan extension. This discrepancy may be due to the different levels of reduction in *rpia-1* expression levels. As the *rpia-1* knockout mutants showed developmental delay (Figure S1C), which did not occur in the *Drosophila* hypomorph mutant, the *rpia-1* null mutation might possess a negative effect on physiological regulation during development. However, *rpia-1* knockdown by feeding *rpia-1* dsRNA-containing bacteria starting at the embryonic stage also failed to prolong lifespan. Thus, instead of feeding bacteria at the embryonic stage, we postponed the onset of knockdown to the post-developmental stage with the purpose of preventing *rpia-1* down-regulation from affecting development. The outcome showed that the knockdown of *rpia-1* after the developmental stage indeed extended lifespan, supporting our hypothesis. The correlation between PPP and development has not been established in animals, whereas PPP has been associated with cancer metabolism and cell survival [25,59,60]. Since PPP is not only involved in glucose catabolism, and the metabolites generated by this process, such as NADPH, also help in detoxifying intracellular reactive oxygen species, PPP plays vital roles in cancer development and survival [61]. A previous study in *Arabidopsis* reported that the dysregulation of PPP disrupts development and that ribose-5-phosphate (which is generated by RPIA) is essential in embryonic development [62]. These findings support our observation that the lack of *rpia-1* during development may disrupt larvae growth and might disrupt the *rpia-1*-mediated longevity effect in *C. elegans*.

Besides the temporal aspects, spatial aspects also mattered in *rpia-1*-mediated longevity. Consistent with our previous findings in *Drosophila* [22]*,* we also observed the highest levels of lifespan extension in worms with neuron-enhanced knockdown of *rpia-1*. In order to in depth examine which specific neuron cell type is the most crucial in *rpia-1*-mediated lifespan regulation, we conducted lifespan experiments in neuron cell-type-specific knockdown strains [44]. Intriguingly, we identified two specific cell types, glutamatergic and cholinergic neurons, which were responsible for longevity regulation. These neurons are also known as excitatory neurons, where the action potential of presynaptic neurons may increase the potential action of postsynaptic neurons. Neuron excitation consumes nearly 80–85% of the ATP generated to pass neural signals; thus, the rates of the TCA cycle and glucose oxidation, which are the major pathways to generate ATP, increase the most in excitatory neurons, especially in glutamatergic neurons [63]. Therefore, reducing *rpia-1* in excitatory neurons sustains the PPP in the oxidative phase and produces fewer non-oxidative-phase metabolites, which might affect the process of excitatory neurotransmission and suppress neuron excitation. The role of excitatory neuron suppression in longevity has been reported by a previous study, where genetic inhibition of neural excitation resulted in lifespan extension in both rodents and worms [64]. Along with our experimental results, we proposed the hypothesis that the down-regulation of *rpia-1* might reduce the production of energy in excitatory neurons and suppress neural excitation by inhibiting metabolic processes in excitatory neurons. This hypothesis is partially supported by our IPA results which indicated a reduction in glutamate receptor signaling (Figure S2B); however, further investigations will be needed to test this hypothesis in the future.

Aside from the knockdown of *rpia-1*, we also generated strains with *rpia-1* overexpression by extrachromosomal array and investigated whether *rpia-1* overexpression may negatively affect longevity. By overexpressing *rpia-1* under its endogenous promoter, we were able to observe lifespan changes that were specifically attributable to *rpia-1* overexpression, without ectopic effects (Figure S5A,B). Overexpression of *rpia-1* showed lifespan reduction in worms (Figure S5C,D). Although the outcome matched our expectations, it did not match our previous findings that *Rpi* overexpression in *Drosophila* did not affect lifespan [22]. This discrepancy might be due to the difference between the promoters. In our previous study, we overexpressed the coding sequence of *Rpi* using ubiquitous and pan-neuronal promoters in *Drosophila*, whereas we overexpressed the genomic sequence of *rpia-1* under its own endogenous promoter in this study. Thus, the ectopic expression and the extent of expression levels may be the main factor leading to this difference.

Our experimental results demonstrated a novel molecular mechanism, besides NADPH elevation, involved in *rpia-1*-mediated longevity effects, that is, the activation of autophagy. The up-regulation of autophagy has been considered beneficial to longevity [10]. Numerous longevity-associated treatments result in increased autophagy, such as caloric restriction, hormetic heat shock, and mitochondrial hormesis [49,65,66]. Moreover, these findings even point out that several longevity-related molecular mechanisms prolong lifespan by activating the AMPK pathway and inhibiting the TOR pathway [67]. The reduction of human RPIA in different cancer cell types, including HeLa cells, pancreatic cancer cells, and lung cancer cells, induces autophagic flux and promotes tumor progression by regulating redox signaling [24,25,68]. Therefore, this evidence prompted us to investigate whether *rpia-1*-mediated longevity also required the induction of autophagy. Our results confirmed our speculation that autophagy induction is involved in *rpia-1* knockdown-mediated lifespan regulation.

As the AMPK and TOR pathways are the major upstream mechanisms regulating autophagy [67] and are associated with cellular energy homeostasis [5], we also wondered if these pathways affected *rpia-1* knockdown-mediated longevity. Both our experimental results and RNA-seq analysis suggested that these two pathways were involved in *rpia-1* knockdown-mediated longevity. Furthermore, this evidence implied that the AMPK pathway might have the most crucial role in this manipulation. Acting as a kinase in the process of balancing ATP and AMP, the major function of AMPK is to manipulate energy homeostasis. The third enzyme of oxidative phase of PPP (oxiPPP), 6-phosphogluconate dehydrogenase (6-PGD), which can produce ribulose-5-phosphate (Ru-5-P) from 6-phosphogluconate (6-PG), has been shown to inhibit AMPK activity through producing Ru-5-P to disrupt LKB1 complex [69]. The knockdown of the second enzyme of oxiPPP, 6-phosphogluconolactonase (PGLS), increases the generation of the byproduct γ-6-phosphogluconolactone (γ-6PGL), which binds to Src to trigger protein phosphatase 2A (PP2A) recruitment and phosphorylation, and eventually causes AMPK activation [70,71].

According to the evidence above, we proposed that *rpia-1* mediates lifespan through two major mechanisms to regulate the activation of the AMPK pathway, that is, the reduction of nucleotide synthesis and the phosphorylation of PP2A (Figure S6) [70]. Previous studies on pancreatic ductal adenocarcinoma have revealed that deregulation of RPIA reduces nucleotide synthesis [72], which triggers activation of the AMPK pathway in response to the low energy status in cells and may benefit longevity [73,74,75]. On the other hand, the deregulation of *Rpi* in our previous work resulted in the activation of G6PD activity [22], which may increase the production of δ-6PGL. While the δ-6PGL is transformed into a byproduct, γ-6PGL, it induces the phosphorylation of PP2A and eventually leads to AMPK pathway activation. Accompanying the activation of AMPK and the preservation of oxiPPP, the NADPH may accumulate, the TOR pathway may be inhibited, and autophagy flux may be enhanced. Nevertheless, this hypothesis requires more supporting evidence in future experiments which may focus on the role of the intervention of oxiPPP-AMPK in longevity.

## 5. Conclusions

In sum, our results support that the longevity effects mediated by *rpia-1* reduction could be evolutionarily conserved. Not only did the knockdown of *rpia-1* enhance oxidative stress tolerance and ameliorate neural polyglutamine toxicity but it also prolonged the lifespan in worms (Figure 8) as well as in flies. Moreover, we also validated the spatial and temporal effects of *rpia-1* knockdown in lifespan regulation. That is, knocking down *rpia-1* ubiquitously in the post-developmental stage or specifically, in excitatory neurons can both extend lifespan. Additionally, we elucidated the molecular mechanisms crucial for *rpia-1* knockdown-mediated lifespan extension, such as the activation of the AMPK pathway and autophagy, or the inhibition of TOR. These findings suggest that *rpia-1* knockdown-mediated longevity is an evolutionarily conserved phenomenon, which may indicate that the regulation of *rpia-1* can be a potential strategy to improve healthspan and ameliorate aging.

## Figures and Tables

**Table 1:** Tissue-specific strain information.

Strain Name	Genotype and Description	Regulated Genes/Longevity Effects	Reference
TU3401	*uIs69 [pCFJ90 (myo-2p::mCherry) + unc-119p::sid-1].* Neuron-hypersensitive knockdown line.	*daf-2*↓/lifespan↑; *daf-16*↓/lifespan↓	[40]
WM118	*neIs9 [myo-3::HA::RDE-1 + rol-6(su1006)].* Muscle-specific knockdown line.	*kin-1*↓/lifespan↑; *kin-2*↓/lifespan↓	[41]
VP303	*kbIs7 [nhx-2p::rde-1 + rol-6(su1006)].* Intestine-specific knockdown line.	*rpc-1*↓/lifespan↑	[42]
NR222	*kzIs9 [(pKK1260) lin-26p::NLS::GFP + (pKK1253) lin-26p::rde-1 + rol-6(su1006)].* Hypodermis-specific knockdown line.	*pry-1*↓/lifespan↓	[43]

**Table 2:** Neuron cell-type-specific strain information.

Strain Name	Genotype and Description	Regulated Genes/Longevity Effects	Reference
XE1582	*wpSi11 [eat-4p::rde-1::SL2::sid-1 + Cbr-unc-119(+)] II*. Glutamatergic neuron-specific knockdown line.	*cbp-1*↓/lifespan↓	[45]
XE1581	*wpSi10 [unc-17p::rde-1::SL2::sid-1 + Cbr-unc-119(+)] II.* Cholinergic neuron-specific knockdown line.		
XE1375	*wpIs36 [unc-47p::mCherry] I. wpSi1 [unc-47p::rde-1::SL2::sid-1 + Cbr-unc-119(+)] II.* GABAergic neuron-specific knockdown line.		
XE1474	*wpSi6 [dat-1p::rde-1::SL2::sid-1 + Cbr-unc-119(+)] II.* Dopaminergic neuron-specific knockdown line.		

## Data Availability

The RNA-seq data can be downloaded at the following website: https://www.ncbi.nlm.nih.gov/geo/query/acc.cgi?acc=GSE216697, with the secure token “gtcdgeagzjitpgb“, accessed on 31 May 2023.

## Supplementary Materials

The following supporting information can be downloaded at https://www.mdpi.com/article/10.3390/antiox12010124/s1. Figure S1. CRISPR knockout of *rpia-1* mutants did not affect lifespan but resulted in developmental delay. Figure S2. IPA analysis provides biological functions and canonical pathways which are affected by knockdown of *rpia-1*. Figure S3. IPA analysis provides bio-functions and canonical pathways affected by knockdown of *rpia-1*. Figure S4. ORA analysis identifies potential down-regulated downstream target genes, which may be involved in *rpia-1* knockdown-mediated longevity effect. Figure S5. Overexpression of *rpia-1* shortens lifespan. Figure S6. Hypothesis of *rpia-1* downregulation mediating longevity through activating AMPK pathway.
