# Supplementary material for: Reduced Ribose-5-Phosphate Isomerase A-1 Expression in Specific Neurons and Time Points Promotes Longevity in Caenorhabditis elegans
Source: Antioxidants (Basel). 2023 Jan 4;12(1):124. doi: 10.3390/antiox12010124 (PMC9854458; doi:10.3390/antiox12010124)
Supplement: Supplementary file 1 [file antioxidants-12-00124-s001.zip › antioxidants-2064546-supplementary.pdf]

Supplementary data

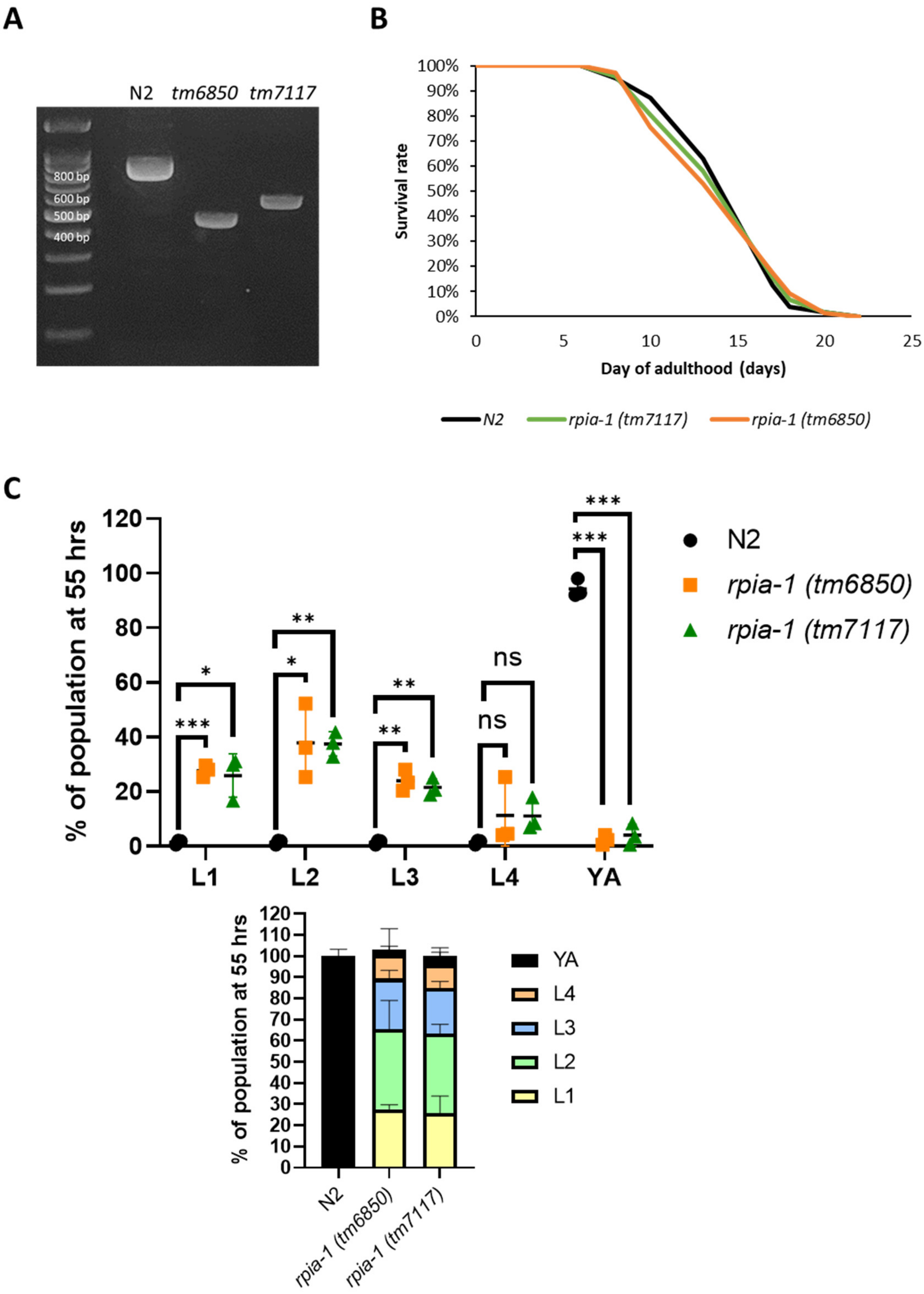

Figure S1. CRISPR knockout of *rpia-1* mutants *tm6850* and *tm7117* did not affect lifespan but exhibit developmental delayed phenotype. (**A**) PCR verification of the two CRISPR knockout mutants showed depletion of *rpia-1* genomic sequence. (**B**) CRISPR knockout mutants displayed no lifespan changes compare to N2 control (N.S., $p > 0.05$., by Log-rank test). (**C**) Both *rpia-1* mutants exhibited developmental delayed phenotype ($p<0.05$ *, $p<0.01$ ** and $p<0.001$ ***. The statistical analysis was conducted by t-test and ANOVA).

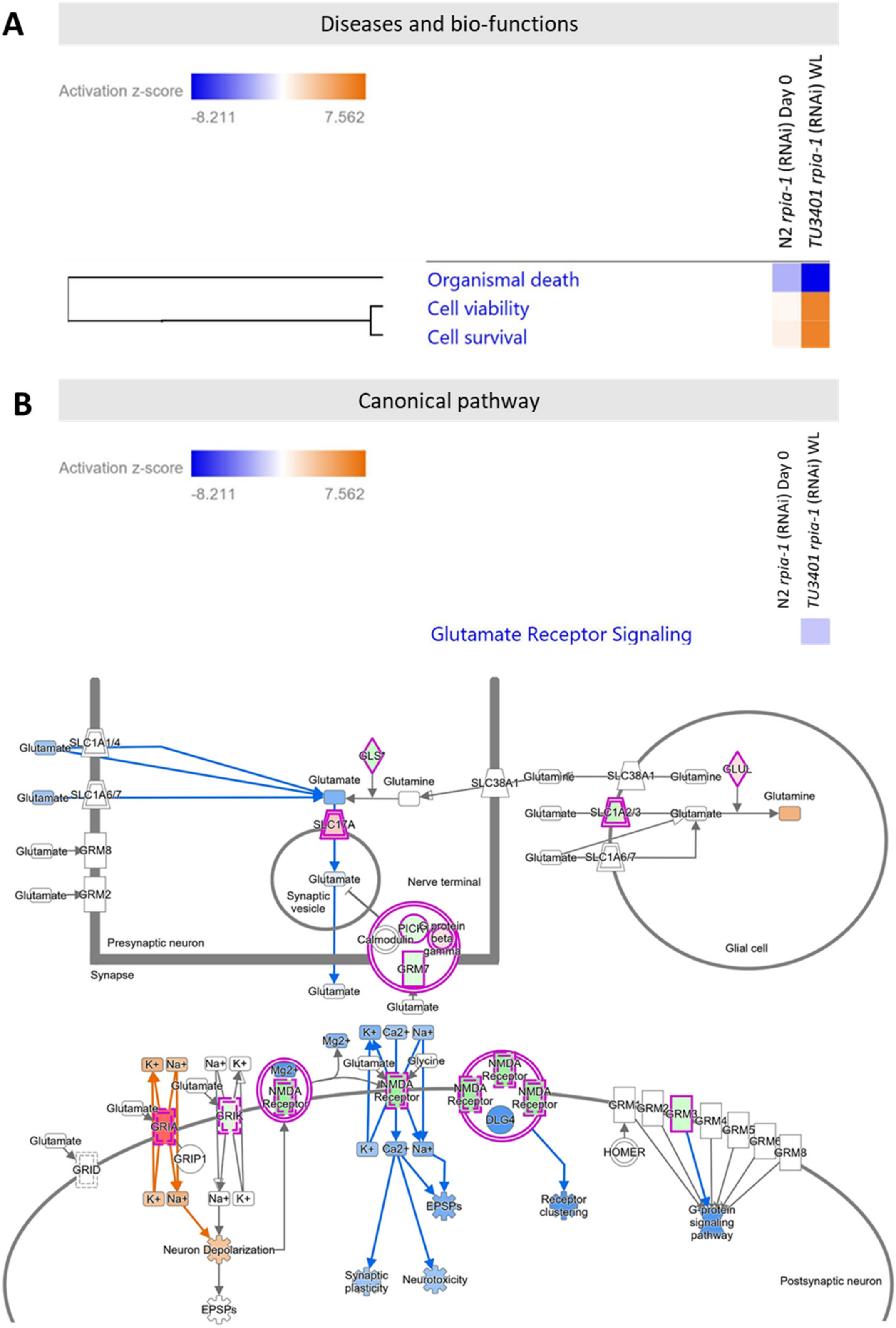

Figure S2. IPA analysis provides biological functions and canonical pathways which are affected by knockdown of *rpia-1*. IPA analysis indicates that (**A**) knockdown of *rpia-1* reduces organismal cell death, increases cell viability. (**B**) Canonical pathway analysis results show that knockdown *rpia-1* in neurons reduced glutamate receptor signaling.

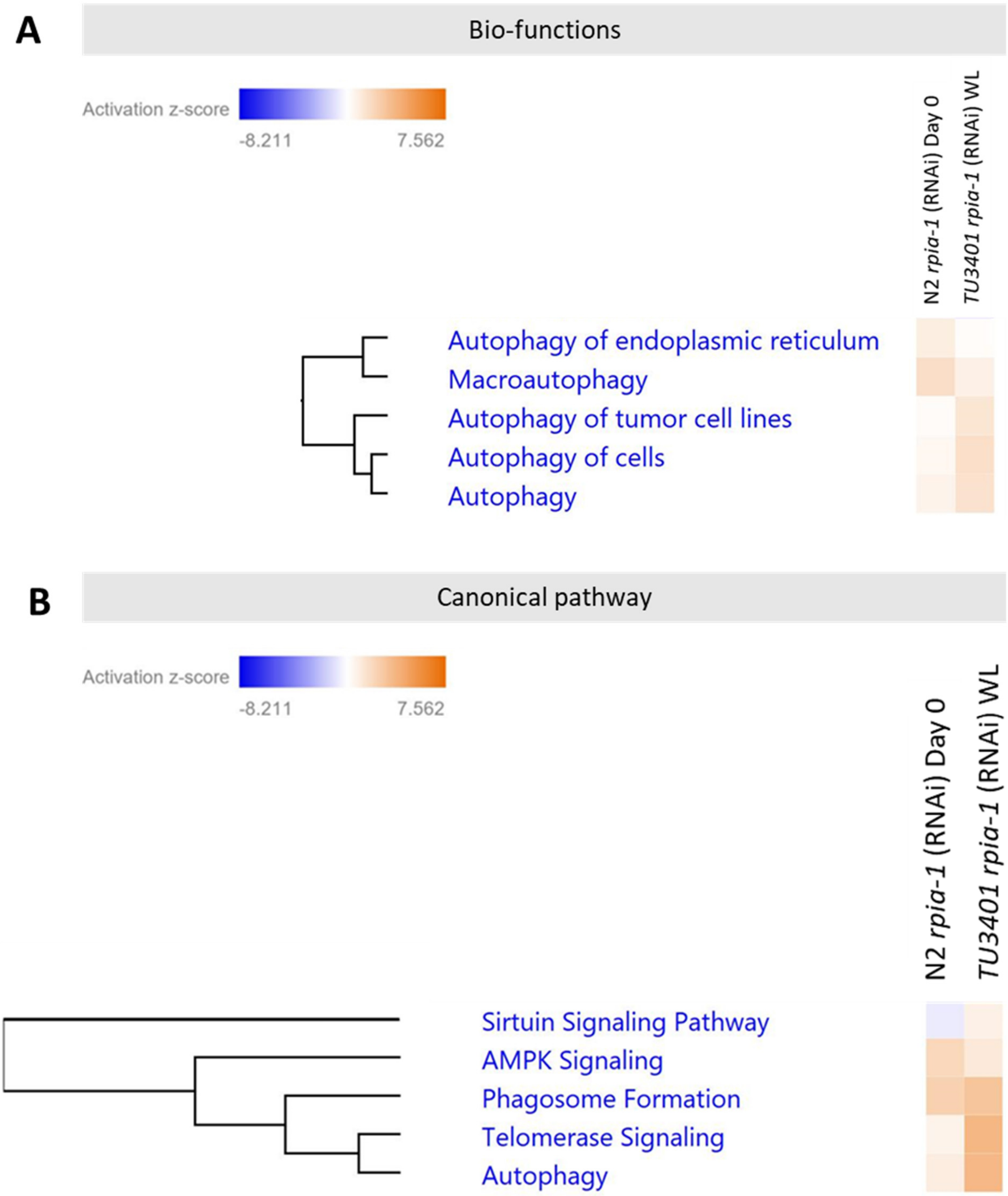

Figure S3. IPA analysis provides bio-functions and canonical pathways which are affected by knockdown of *rpia-1*. (**A**) IPA analysis results supported our experimental findings that knockdown of *rpia-1* induces autophagy-related bio-function. (**B**) Canonical pathway analysis shows that several longevity-related canonical pathways are activated by *rpia-1* knockdown.

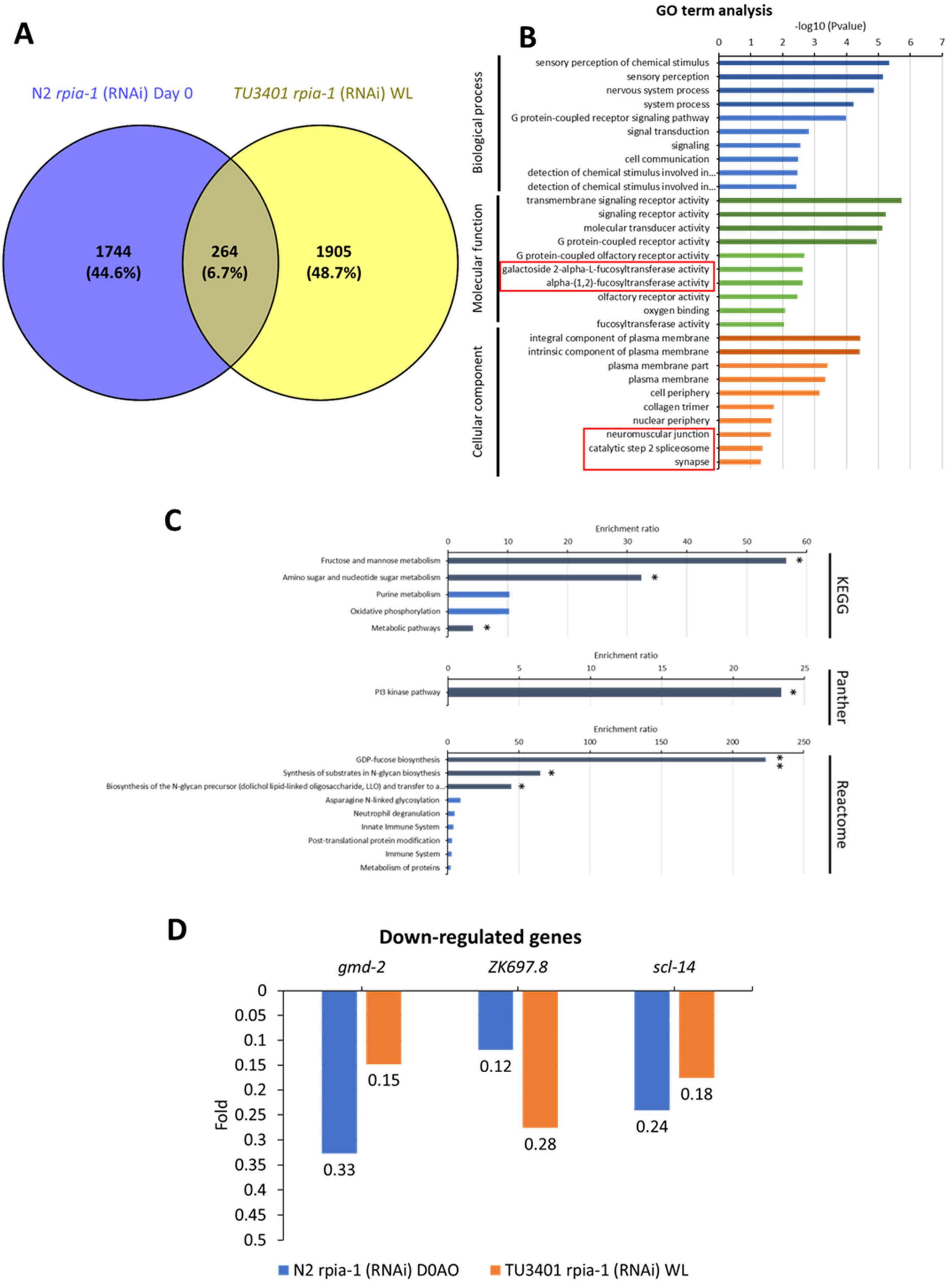
A
N2 rpia-1 (RNAi) Day 0
TU3401 rpia-1 (RNAi) WL
1744 (44.6%)
264 (6.7%)
1905 (48.7%)
B
GO term analysis
-log10 (Pvalue)
0 1 2 3 4 5 6 7
Biological process
sensory perception of chemical stimulus
sensory perception
nervous system process
system process
G protein-coupled receptor signaling pathway
signal transduction
signaling
cell communication
detection of chemical stimulus involved in...
detection of chemical stimulus involved in...
Molecular function
transmembrane signaling receptor activity
signaling receptor activity
molecular transducer activity
G protein-coupled receptor activity
G protein-coupled olfactory receptor activity
galactoside 2-alpha-L-fucosyltransferase activity
alpha-(1,2)-fucosyltransferase activity
olfactory receptor activity
oxygen binding
fucosyltransferase activity
Cellular component
integral component of plasma membrane
intrinsic component of plasma membrane
plasma membrane part
plasma membrane
cell periphery
collagen trimer
nuclear periphery
neuromuscular junction
catalytic step 2 spliceosome
synapse
C
Enrichment ratio
0 10 20 30 40 50 60
Fructose and mannose metabolism *
Amino sugar and nucleotide sugar metabolism *
Purine metabolism
Oxidative phosphorylation
Metabolic pathways *
KEGG
Enrichment ratio
0 5 10 15 20 25
PI3 kinase pathway *
Panther
Enrichment ratio
0 50 100 150 200 250
GDP-fucose biosynthesis **
Synthesis of substrates in N-glycan biosythesis *
Biosynthesis of the N-glycan precursor (dolichol lipid-linked oligosaccharide, LLO) and transfer to a... *
Asparagine N-linked glycosylation
Neutrophil degranulation
Innate Immune System
Post-translational protein modification
Immune System
Metabolism of proteins
Reactome
D
Down-regulated genes
gmd-2
ZK697.8
scl-14
Fold
0 0.05 0.1 0.15 0.2 0.25 0.3 0.35 0.4 0.45 0.5
0.33
0.15
0.12
0.28
0.24
0.18
N2 rpia-1 (RNAi) D0AO
TU3401 rpia-1 (RNAi) WL

Figure S4. ORA analysis identifies potential down-regulated downstream target genes which may be involved in *rpia-1*-mediated longevity**.** The preprocessing procedure of conducting ORA analysis followed the description mentioned in figure 7. (**A**) 264 genes were downregulated in worms with both ubiquitous and pan-neuronal knockdown of *rpia-1*, and these 264 genes were applied to conduct further ORA analysis by WebGestalt. For (**B-C**), the data presented is log transformed p-value (FDR corrected) of GO terms or pathways found to be enriched in the up-regulated gene set. (**B**) GO term analysis revealed the gene classes with significant changes. The red frames indicate the classes which were specifically found among the down-regulation gene sets. (**C**) Pathway analysis results including KEGG, Panther, and Reactome. Each pathways-analysis showed the enrichment ratio of the pathways. The bars with darker color indicated the gene set of pathways was significantly induced. (*, $p < 0.05$; **, $p < 0.01$; ***, $p < 0.001$. The statistical analysis was conducted by Fisher exact test). (**D**) The gene identified by GO-term analysis. (**E**) The genes identified by GO-term analysis. Blue graph indicated fold changes of gene expression level in ubiquitously knockdown strain. In (**D**) and (**E**), blue bars indicate changes in gene expression levels upon ubiquitous knockdown; orange bars indicate those upon pan-neuronal knockdown.

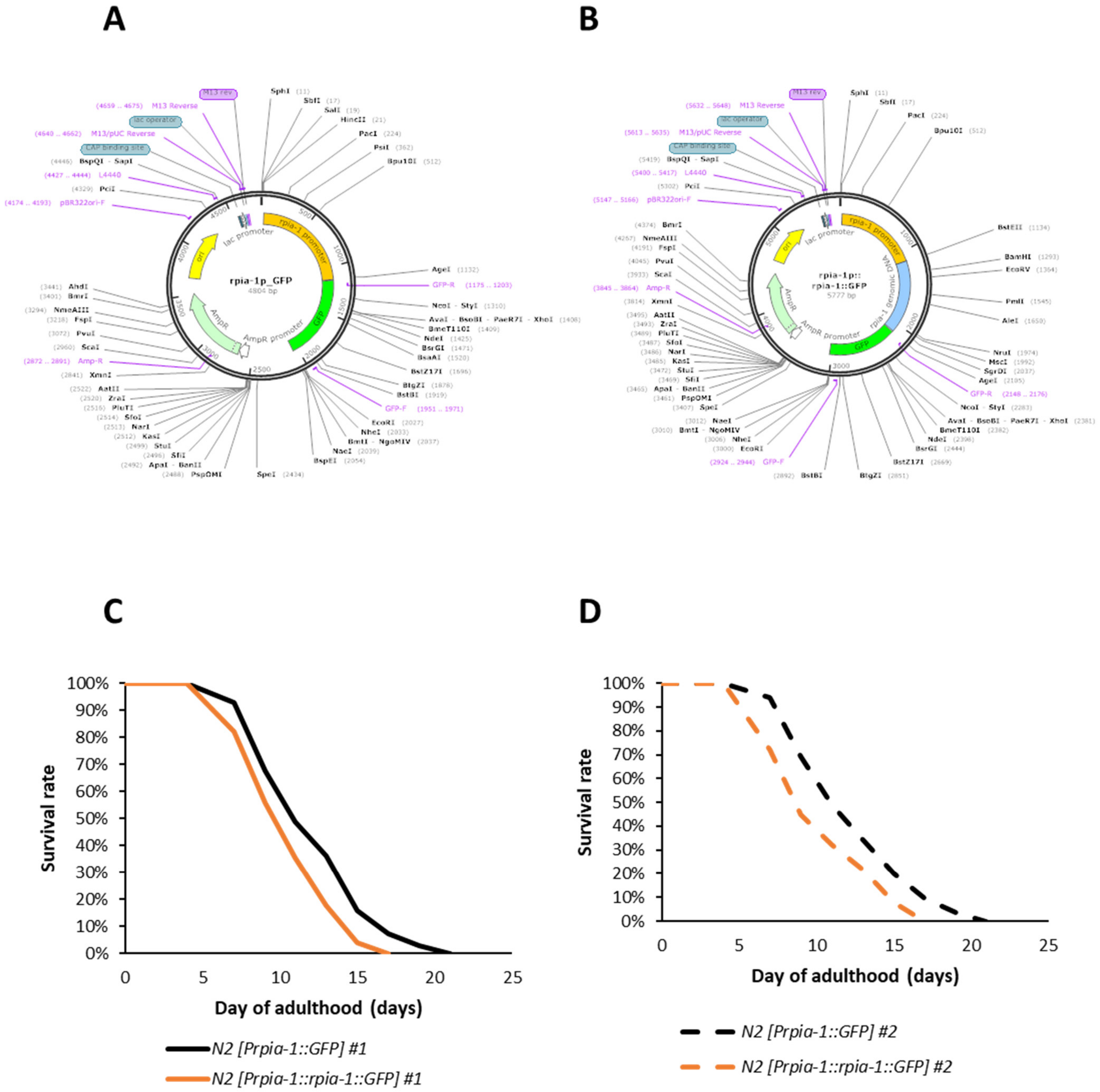

Figure S5. Overexpression of *rpia-1* shortens lifespan in *C. elegans*. (**A**, **B**) The construct maps of *rpia-1* endogenous promoter driving GFP as control (*Prpia-1::GFP*) and driving *rpia-1* fused with *GFP* as overexpression (*Prpia-1::rpia-1::GFP*). (**C**, **D**) Two independent *rpia-1* overexpression strains (solid and dashed orange lines, *Prpia-1::rpia-1::GFP #1 and #2*) showed reduction of 11.4% and 13.8% in mean lifespan compared to their own controls (solid and dashed black lines, *Prpia-1::GFP #1 and #2*) (***, $p < 0.001$, by log-rank test).

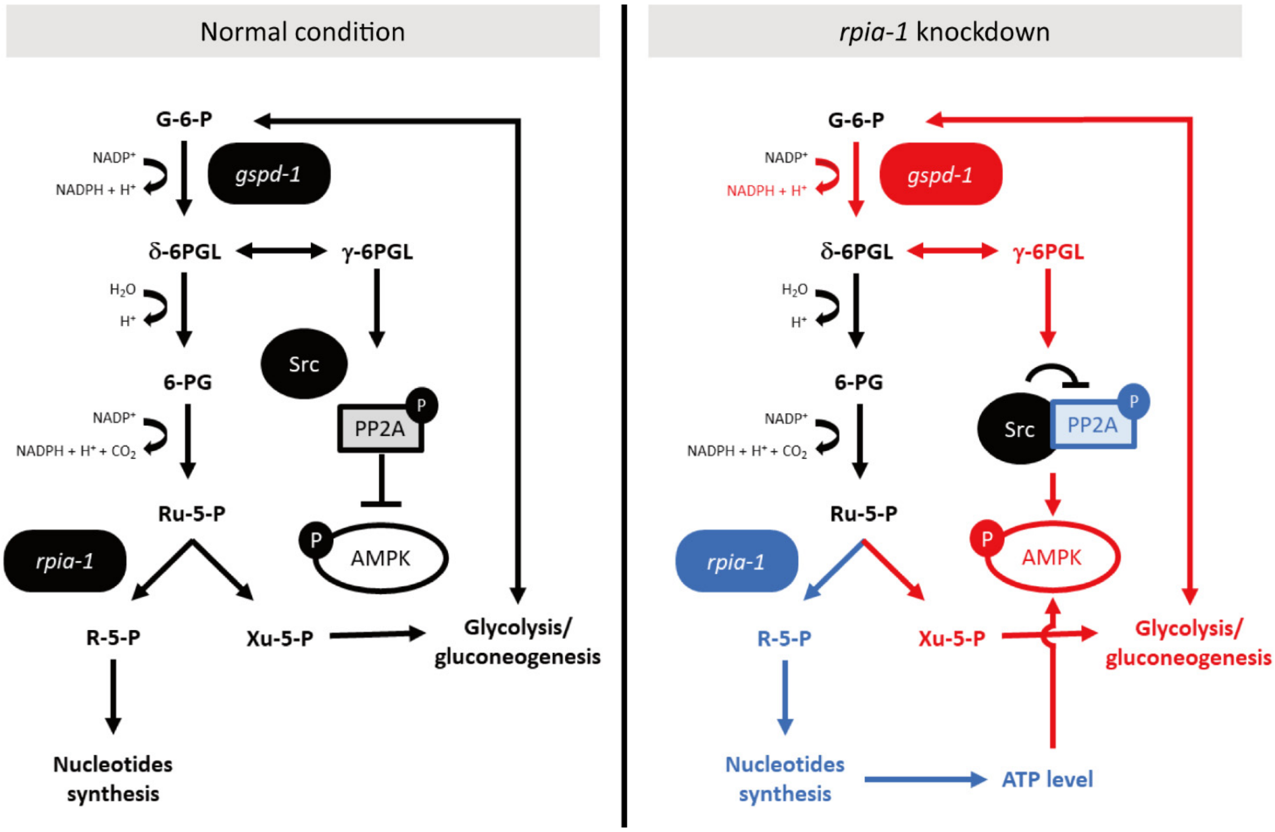

Figure S6. Hypothesis of *rpia-1* downregulation mediating longevity through activating AMPK pathway. (**Left**) The oxidative phase of PPP produces NADPH through the process of converting glucose-6-phosphate (G-6-P) to ribulose-5-phosphate (Ru-5-P) under normal conditions. Once Ru-5-P is converted to ribose-5-phosphate (R-5-P), the PPP enters the nonoxidative phase and generates nucleotides. On the other hand, the protein phosphatase 2A (PP2A) without phosphorylation inhibits AMPK phosphorylation to disrupt the activation of AMPK pathway. (**Right**) The reduction of *rpia-1* may suppress nucleotide synthesis and sustain PPP in oxidative phase, which may increase NADPH production and induce *gspd-1* expression to convert G-6-P into δ-6-phosphogluconolactone (δ-6PGL). The intramolecular rearrangement of δ-6PGL generates γ-6-phosphogluconolactone (γ-6PGL), which binds to Src and enhances PP2A recruitment. This process triggers the phosphorylation of PP2A, which inhibits the activity of PP2A, and eventually contribute to activation of AMPK pathway. In addition, suppression of nucleotide synthesis may lead to a reduction in ATP levels, which triggers AMPK activity to increase catabolism.
